# Supplementary material for: Processes of care and survival associated with treatment in specialist teenage and young adult cancer centres: results from the BRIGHTLIGHT cohort study
Source: BMJ Open. 2021 Apr 7;11(4):e044854. doi: 10.1136/bmjopen-2020-044854 (PMC8031022; doi:10.1136/bmjopen-2020-044854)
Supplement: Supplementary data [file bmjopen-2020-044854supp001.pdf]

Supplemental file

Figure A1: Matrix representing the causal diagram explaining confounding variable to enter in the Directed Acyclic Graph (DAG)

	TYA CoC	Survival	Age	Gender	CS	CT	SES	Ethnicity	Geography	DoH	Treatment	RtD	QOL
TYA CoC		⇗	⇙	⇙	⇙	⇙	⇙	⇙	⇙	O	⇙	⇙	⇗
Survival			⇙	⇙	⇙	⇙	⇙	⇙	⇙	⇙	⇙	⇙	⇙
Age				O	⇗	⇗	⇗	O	O	⇗	⇗	⇗	⇗
Gender					⇗	⇗	O	O	O	⇗	⇗	⇗	⇗
CS						⇙	⇙	⇙	⇙	⇗	⇗	⇙	⇗
CT							⇙	⇙	⇙	⇗	⇗	⇗	⇗
SES								⇙	⇙	⇗	⇗	⇗	⇗
Ethnicity									⇙	⇗	⇗	⇗	⇗
Geography										⇗	⇗	⇗	⇗
DoH											⇙	⇙	⇗
Treatment												⇙	⇗
RtD													⇗
QOL													

CS: cancer severity; CT: cancer type; DoH: duration of hospitalisation; QOL: quality of life; RtD: route to diagnosis; SE: symptom experience; SES: socioeconomic status; SS: social support; TYA CoC: teenage and young adult category of care

O – indicates a null relationship

Fern LA, *et al. BMJ Open* 2021; 11:e044854. doi: 10.1136/bmjopen-2020-044854
